# Age-Related Hearing Loss Is Accompanied by Chronic Inflammation in the Cochlea and the Cochlear Nucleus

**DOI:** 10.3389/fnagi.2022.846804

**Published:** 2022-03-28

**Authors:** Benjamin J. Seicol, Shengyin Lin, Ruili Xie

**Affiliations:** ^1^Department of Otolaryngology—Head and Neck Surgery, The Ohio State University, Columbus, OH, United States; ^2^Department of Neuroscience, The Ohio State University, Columbus, OH, United States

**Keywords:** aging, macrophage, inflammation, hearing loss, cochlea, cochlear nucleus, microglia, complement

## Abstract

Age-related hearing loss (ARHL) is a major hearing impairment characterized by pathological changes in both the peripheral and central auditory systems. Low-grade inflammation was observed in the cochlea of deceased human subjects with ARHL and animal models of early onset ARHL, which suggests that inflammation contributes to the development of ARHL. However, it remains elusive how chronic inflammation progresses during normal aging in the cochlea, and especially the accompanying changes of neuroinflammation in the central auditory system. To address this, we investigated chronic inflammation in both the cochlea and the cochlear nucleus (CN) of CBA/CaJ mice, an inbred mouse strain that undergoes normal aging and develops human, like-late-onset ARHL. Using immunohistochemistry, confocal microscopy, and quantitative image processing, we measured the accumulation and activation of macrophages in the cochlea and microglia in the CN using their shared markers: ionized calcium binding adaptor molecule 1 (Iba1) and CD68—a marker of phagocytic activity. We found progressive increases in the area covered by Iba1-labeled macrophages and enhanced CD68 staining in the osseous spiral lamina of the cochlea that correlated with elevated ABR threshold across the lifespan. During the process, we further identified significant increases in microglial activation and C1q deposition in the CN, indicating increased neuroinflammation and complement activation in the central auditory system. Our study suggests that during normal aging, chronic inflammation occurs in both the peripheral and the central auditory system, which may contribute in coordination to the development of ARHL.

## Introduction

Age-related hearing loss (ARHL) is among the most common ailments in the aging population, affecting one in three people over the age of 60 and greater than one in two people over the age of 75 (Lin et al., 2011). Its prevalence is expected to continue to climb given an increasing aging population (US Census Bureau, 2014). Traditionally, ARHL was classified into different types based on distinctive pathological changes of the cochlea and the altered audiogram (Schuknecht, 1964; Johnsson and Hawkins, 1972; Schuknecht and Gacek, 1993). The most prominent changes are the loss of sensory hair cells and spiral ganglion neurons (SGNs; Makary et al., 2011). Recent studies showed that long before the substantial cell loss and profound hearing threshold elevation, cochlear synapses that connect hair cells and SGNs can be significantly damaged (Kujawa and Liberman, 2015; Liberman, 2017; Kohrman et al., 2020) and may be the onset-contributor of ARHL (Stamataki et al., 2006; Sergeyenko et al., 2013; Liberman, 2017; Liberman and Kujawa, 2017). Deafferentation at the cochlear synapses during aging is accompanied by the correspondingly degenerated central auditory nerve (AN) synapses (O’Neil et al., 2011; Wang et al., 2021), and collectively lead to reduced auditory input to the cochlear nucleus (CN; Xie, 2016; Xie and Manis, 2017; Wang et al., 2021) that impacts the signal processing of the entire central auditory system and results in perceptual deficits of ARHL. Treatments for ARHL primarily involve increasing sensory input to the auditory system through medical devices such as hearing aids or cochlear implants. Interventions to prevent the progression or restore lost function remain lacking, partially due to an incomplete understanding of the mechanisms underlying human-like ARHL in animal models (Bowl and Dawson, 2015). The question can be better investigated in animal models that undergo normal aging and develop late-onset ARHL mimicking human conditions.

Inflammation, or broadly, the activation of innate immune responses, is a common pathway through which early-life insults can cause long-term chronic pathology in peripheral and central neural tissues (Finch, 2011). Macrophages in the cochlea and microglia in the brainstem are long-lived resident innate immune cells that can promote as well as resolve acute inflammation in their respective niches depending on their state of activation (Mosser and Edwards, 2008; Bachiller et al., 2018; Liu et al., 2018). Failure to resolve inflammation may contribute to cochlear pathology including synaptopathy and hair cell loss. For example, cochlear macrophages were recently found to accumulate in the AN in patients with ARHL (Noble et al., 2019), whereas fully differentiated macrophages in the basilar membrane were dynamically activated in response to hair cell degeneration in C57 mice with early onset ARHL (Frye et al., 2017). Ionized calcium binding adaptor molecule 1 (Iba1) is a cytoskeletal protein expressed in resident macrophages and is a useful marker to identify both cochlea macrophages and microglia to measure their accumulation and changes in cellular morphology associated with increased activation (Ohsawa et al., 2004; Hovens et al., 2014; Bennett et al., 2016). CD68 is a marker that reflects phagocytic activity in both macrophages and microglia, and the level of CD68 corresponds with pro-inflammatory activation (Holness and Simmons, 1993; Ramprasad et al., 1996; Papageorgiou et al., 2016; Chistiakov et al., 2017; Zrzavy et al., 2017). Using these tools, macrophages have been shown to reside throughout the cochlea and likely perform different functions depending on location given a wide variation in morphologies (Liu et al., 2018). Macrophages in the osseous spiral lamina (OSL) are of particular interest because of their close proximity to cochlear synapses and adjacency with AN fibers. Since the bony labyrinth constrains the amount of swelling possible in this area, a small change in accumulation or activation of OSL macrophages could have dramatic impacts on auditory function. The active processes involved in the resolution of inflammation following noise-, drug-, or age-induced sensorineural hearing loss are likely relatively conserved (Kalinec et al., 2017) and studies on these various insults all support the notion that promoting the pro-resolution activation of macrophages is an exciting target for preventing the progression of sensorineural hearing loss. In particular, age-related inflammation, which is often called “inflammaging”, uniquely results from the immunosenescence of the adaptive immune system and compensatory innate immune activation which partially restores host defense functions (Franceschi et al., 2000; Watson et al., 2017). Inflammaging during normal aging likely contributes to ARHL in humans (Verschuur et al., 2014). Therefore, it is critical to study animal models of late-onset ARHL with carefully controlled age groups and hearing status.

In the central nervous system, neuroinflammation contributes to the progression of numerous age-related conditions, including cognitive decline in Alzheimer’s disease (Hong et al., 2016a) and sensory loss in the aging visual and auditory cortices (Tremblay et al., 2012). Microglial activation can be caused by decreased neuronal activity (Schafer et al., 2012) and result in increased complement deposition on tissues during aging (Stephan et al., 2013). While microglial activation and complement deposition have been investigated in the CN during development (Noda et al., 2019), the dynamics of age-related changes in CN microglia and complement remain unclear. To address these gaps, we investigated chronic inflammation in the cochlea and CN of CBA/CaJ mice with human-like, late-onset ARHL (Sergeyenko et al., 2013; Wang et al., 2021). We investigated cochlear macrophage accumulation and activation as well as microglial activity and complement deposition in the CN of young (1.5–4.5 months), middle-aged (17–19 months), and aged (28–32 months) CBA/CaJ mice of either sex, using immunohistochemistry, confocal microscopy, and quantitative image analysis. The hearing status of all mice was assessed by auditory brainstem response (ABR) to quantify the hearing loss. We found a progressive increase in age-associated inflammation in the cochlea and CN during ARHL corresponding with increased ABR threshold, suggesting that chronic inflammation occurs in both the peripheral and central auditory systems and may contribute in coordination to the development of ARHL. In particular, the enhanced inflammation in middle-aged mice at the early stage of ARHL suggests that there might be a time window during aging for earlier detection and development of novel therapeutic targets to prevent the progression of ARHL.

## Materials and Methods

### Ethical Approval

All experiments were conducted under the guidelines of the protocols approved by the Institutional Animal Care and Use Committee of The Ohio State University (IACUC protocol number: 2018A00000055), which maintains an Animal Welfare Assurance (#D16-00168/A3261-01) in compliance with the United States Public Health Service Policy on Humane Care and Use of Laboratory Animals. We understand the ethical principles under which the journal operates, and our work complies with the animal ethics checklist.

### Animals

#### Original Source

The study used CBA/CaJ mice of either sex, which were purchased from The Jackson Laboratory (Bar Harbor, ME, USA), bred, and maintained at the animal facility at The Ohio State University (Columbus, OH, USA). Three age groups of mice were used that included 9 young (1.5–4.5 months), 12 middle-aged (17–19 months), and 15 old (28–32 months) mice.

#### Husbandry

All mice were maintained under a 12:12-h light/dark cycle and had ad libitum access to food and water. Auditory levels were measured inside the facility and the noise level in the room was less than 70 dB in broadband, and lower than 30 dB SPL at 10 kHz.

#### Auditory Brainstem Response (ABR)

Mice were deeply anesthetized with an I.P. injection of ketamine (100 mg/kg) and xylazine (10 mg/kg), which was verified by the absence of toe-pinch reflex. The hearing status of the mice was assessed using ABR recordings to determine the lowest decibel level that generates a reliable response to clicks as described previously (Wang et al., 2021). Briefly, anesthetized mice were placed in a sound-attenuating chamber for recording. A feedback-controlled heating pad was used to maintain body temperature at ~36°C. ABR to clicks were recorded on a RZ6-A-P1 system with BioSigRZ software using needle electrodes placed at the ipsilateral pinna and vertex, with the ground electrode at the rump (Tucker-Davis Technologies, Alachua, FL, USA). Clicks (0.1 ms, monophasic with alternating phase; 21 times/s) were delivered through a free field MF1 magnetic speaker (Tucker-Davis Technologies, Alachua, FL, USA) located 10 cm away from the pinna. ABR at each sound level was repeated 512 times and averaged.

### Tissue Isolation and Preservation

Under deep ketamine/xylazine anesthesia, mice were decapitated, and the skulls were opened to retrieve both temporal bones and the brainstem for subsequent processing. Each cochlea was carefully dissected out of the temporal bone as previously described (Fang et al., 2019), flushed with 4% paraformaldehyde (PFA) in 0.1 M phosphate buffered saline (PBS). Cochleae were fixed overnight in 4% PFA in PBS at 4°C. Cochleae were then decalcified using 0.12 M EDTA in 0.1 M PBS for 2–3 days with solution exchanged at least once. *Whole mount cochleae* were prepared as previously described (Fang et al., 2019). Briefly, cochlea preparations (containing the middle and basal turns) were dissected out of the labyrinth under a dissection microscope in 0.1 M PBS. Free floating, whole mount preparations were then stained by IHC as detailed below. For the *brainstems*, parasagittal slices containing the CN were cut at a thickness of 500–600 μm to ensure capture of the entire CN using a Vibratome 1000 (Technical Products, Inc., Cleveland, OH, USA) or a VT1200S Microtome (Leica Biosystems, Wetzlar, Germany). The tissue was then immediately fixed in 4% PFA for 15 min at room temperature and rinsed in PBS (three times for 15 min each). CN tissue was cryoprotected using 30% sucrose in PBS, and then embedded in Cryo-Gel (Cat. #: 475237; Instrumedics Inc., Ann Arbor, MI, USA). Finally, 30 μm thick CN slices were cut using a cryostat slicer (Leica CM3050 S, Leica Biosystems, Deer Park, IL, USA), attached to electrostatic microscope slides, and stored at −80°C until they were processed for immunostaining as described previously (Lin and Xie, 2019).

### Immunohistochemistry

#### Whole Mount Cochlea Preparation

After tissue preparation, whole-mount cochlea tissue was stained as free-floating sections in a 12-well plate. The tissue sections were blocked using 2% BSA, 0.1% Triton X-100 in 0.1 M PBS for 1 h at room temperature. Following blocking, primary antibodies against CD68 (Rat anti-CD68; catalog no. MCA1957; Bio-Rad, Hercules, CA, USA; dilution 1:200), Iba1 (guinea-pig anti-Iba1; catalog no. 234004; Synaptic Systems, Göttingen, Germany; dilution 1:500), and Myosin-VIIA (Rabbit anti-myosin-VIIA IgG; catalog no. 25-6790; Proteus Biosciences, Ramona, CA, USA; dilution 1:500) were applied overnight at 4°C in 1% BSA, 0.1% Triton X-100 in 0.1 M PBS. Sections were then rinsed three times with 0.1 M PBS for 15 min at room temperature, then secondary antibodies (donkey anti-mouse IgG, Alexa Fluor 488, catalog no. A21202, Thermo Fisher Scientific, Waltham, MA, USA; dilution 1:500; goat anti-rat IgG, Alexa Fluor 594, catalog no. A11007; Thermo Fisher Scientific, Waltham, MA, USA; dilution 1:500; goat anti-guinea pig IgG, Alexa Fluor 647 conjugated, catalog no. A21450; Thermo Fisher Scientific, Waltham, MA, USA; dilution 1:500; goat anti-Rabbit IgG, Alexa Fluor 750, catalog no. A21039, Thermo Fisher Scientific, Waltham, MA, USA; dilution 1:500) were applied overnight at 4°C in 1% BSA, 0.1% Triton X-100 in 0.1 M PBS.

#### Cochlear Nucleus Brain Slices

CN slices on slides were incubated in blocking solution (10% horse serum, 0.5% Triton X-100 and 0.05% NaN_3_ in PBS) for 6 h at room temperature. Primary antibodies against Iba1 (guinea-pig anti-Iba1; catalog no. 234004; Synaptic Systems, Göttingen, Germany; dilution 1:500), CD68 (Rat anti-CD68; catalog no. MCA1957; Bio-Rad, Hercules, CA, USA; dilution 1:200), and P2Y12 (rabbit anti-P2Y12; catalog no. AS-55043A; ANASPEC, Fremont, CA, USA; dilution 1:500) or C1q (rabbit anti-C1q, catalog no. ab182451; Abcam, Cambridge, MA, USA; dilution 1:100) were incubated overnight at 4°C. Slides were washed again in PBS (four times for 20 min each). Secondary antibodies (goat anti-guinea pig IgG, Alexa Fluor 647 conjugated, catalog no. A21450; Thermo Fisher Scientific, Waltham, MA, USA; dilution 1:500; goat anti-rat IgG, Alexa Fluor 594, catalog no. A11007; Thermo Fisher Scientific, Waltham, MA, USA; dilution 1:500; goat anti-rabbit IgG, Alexa Fluor 488, catalog no. A32731; Thermo Fisher Scientific, Waltham, MA, USA; dilution 1:500) were applied to the slides at room temperature for 4 h, washed with PBS, and mounted on a slide with DAPI-Fluoromount-G mounting media (Southern Biotech, Birmingham, AL, USA).

### Imaging

All tissues were imaged using an FV3000 confocal microscope (Olympus, Tokyo, Japan). Wide-view images were collected using a 10× air objective, a z-step of 2 μm, and a resolution of 1,024 × 1,024. High-magnification images were collected using a 60× oil immersion objective with no digital zoom, z-steps of 1 μm, and resolution of 1,024 × 1,024.

### Image Analysis

Image analysis was conducted using ImageJ (U. S. National Institutes of Health, Bethesda, MD, USA) software to quantify stained area within regions of interest (ROI), and mean fluorescent intensities for z-stack volumetric images. Briefly, volumes were reduced to 2D maximum projections and individual channels were quantified as appropriate, e.g., stained area. Colocalization analyses were conducted using ImageJ. Complement immunoreactivity was quantified by mean fluorescence intensity and reported in fold change compared to young mice (Stephan et al., 2013; ImageJ).

### Statistics

The study was cross-sectional using three different age groups of mice. Power analyses calculated based on pilot studies showed a sample size of five yields an alpha of 0.05 with a power of >90%. All data groups were tested for normality using Shapiro-Wilk normality tests. Data that satisfied the normality assumptions, including cochlear macrophage accumulation and activation, were compared across all ages using one-way ANOVA to determine significance with *post hoc* Tukey multiple comparisons to address individual group differences. Non-parametric testing, specifically Kruskal-Wallis tests and Dunn’s multiple comparisons tests were performed on data that did not pass normality testing. Linear regression was applied, and Pearson’s correlation coefficient was calculated to quantify the strength of the correlation between the Iba1-labeled area and ABR threshold. All data are presented as the mean ± SE.

## Results

### OSL Macrophage Area Correlates With the Severity of Hearing Loss Across the Lifespan

**Figure 1:**
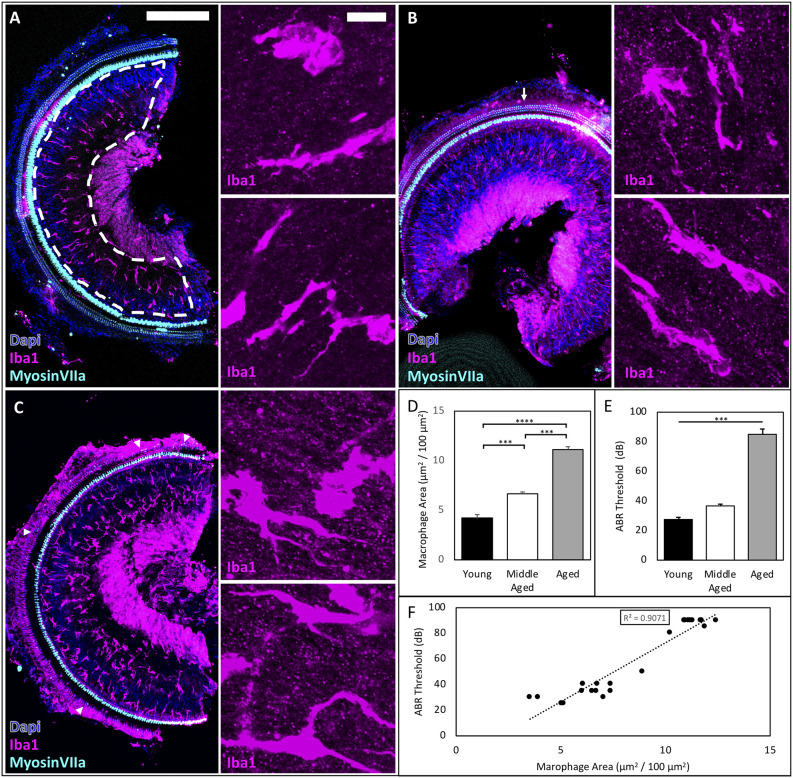
ARHL-associated inflammation increases across the lifespan and correlates with the severity of hearing impairment in CBA/CaJ mice. **(A–C)** Representative images of young, middle-aged, and aged mice showing accumulation of cochlear macrophages in the OSL, scale bar = 200 μm, inserts scale bar = 10 μm. Myosin VIIA staining revealed minimal OHC loss (arrows) in middle-aged mice, while few OHC remained (triangles) in aged mice. **(D)** Significant differences were found between all age groups. ***p < 0.001, ****p < 0.0001. **(E)** ABR Threshold increases with age confirming late-onset ARHL. ***p < 0.001. **(F)** Regression analysis between macrophage area (μm^2^/100 μm^2^) and ABR Threshold to clicks (dB) reveals a significant linear correlation *R*^2^ = 0.9081, *p* < 0.002.

We investigated whether and how macrophages accumulate in the OSL of the cochlea across aging in CBA/CaJ mice. Macrophages coordinate innate immune responses throughout the body, including in the OSL (Brown et al., 2017). The OSL is a thin bony structure in proximity to the organ of Corti that houses fiber bundles including the peripheral projections from SGNs and olivocochlear efferents from the brainstem. We investigated macrophage accumulation and activation in the OSL and found increases during aging (Figure 1). Representative images from young, middle-aged, and aged mice are shown in Figures 1A–C. Macrophage morphology is heterogeneous within the OSL throughout the lifespan, even during homeostasis in young animals (Figure 1A inserts), and this heterogeneity is relatively conserved in middle-aged and aged mice (Figures 1B,C inserts). We therefore analyzed the number and average area of the Iba1-labeled macrophages and found that both were significantly increased in the OSL during aging (macrophage number: young = 259 ± 10.5, middle-aged = 368 ± 21.8, aged = 483 ± 47.7 cells/mm^2^; *n* = 5; one-way ANOVA: *F*_(2,12)_ = 13.3, *p* < 0.001; average macrophage area: young = 164 ± 15.3, middle-aged = 181 ± 7.2, aged = 259 ± 35.9 μm^2^; *n* = 5; one-way ANOVA: *F*_(2,12)_ = 4.875, *p* < 0.05). Given the heterogeneity in morphology and the increase of both macrophage number and average area, our subsequent analyses focused on the total area covered by OSL macrophages. We found that Iba1-labeled area in the OSL increased across aging (Figure 1D; Iba1 area: Young = 4.19 ± 0.42, Middle-aged = 6.66 ± 0.18, Aged = 11.12 ± 0.28 μm^2^/100 μm^2^; *n* = 5, 8, 12, respectively; one-way ANOVA: *F*_(2,21)_ = 137.1, *p* < 0.0001; Tukey multiple comparisons test: *p* < 0.001). We also confirmed the characteristic late-onset of ARHL in CBA/CaJ mice shown in Figure 1E (ABR threshold means: Young = 28 dB, Middle-aged = 37 dB, Aged = 85 dB; one-way ANOVA:*F*_(2,21)_ = 108.9, *p* < 0.0001). The results showed that the Iba1 area correlates with the severity of hearing loss across the lifespan (Figure 1F; *R*^2^ = 0.9081; *p* < 0.002). Remarkably, changes in resident macrophages manifest in the OSL in the early stages of ARHL in the middle-aged mice group, prior to significant tissue damage and cellular loss. This indicates that the changes we observed, including the increase in the Iba1-labeled macrophage area in the OSL, are not a response to the severe tissue degeneration that occurs in late-stage ARHL. Given the early elevation in the macrophage area, we further wanted to understand the functional activation of resident macrophages during normal aging.

### Cochlear Macrophages Upregulate CD68 Indicating Increased Phagocytic Activation

**Figure 2:**
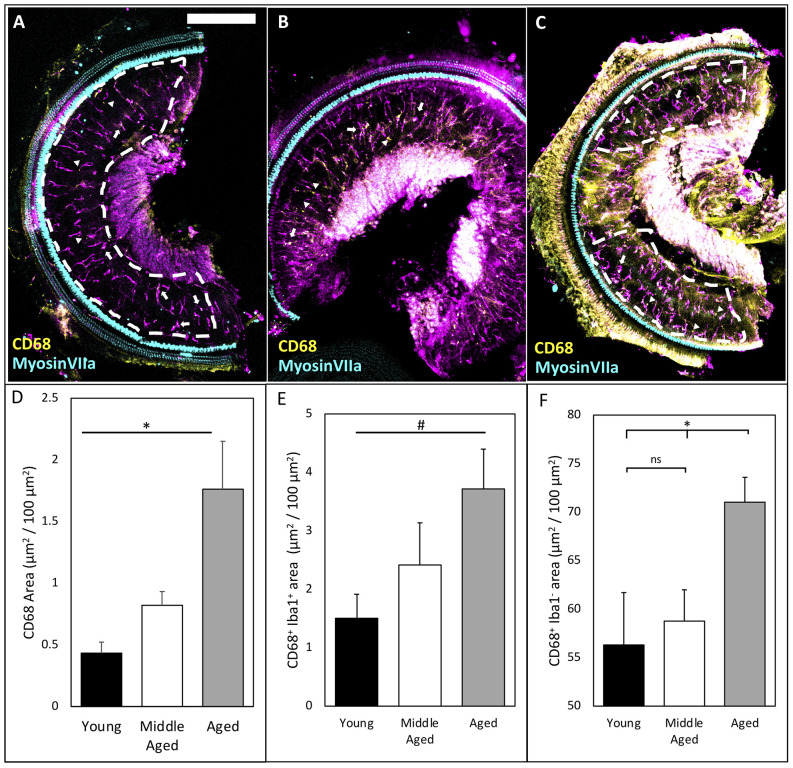
CD68 levels increase in OSL across aging. **(A–C)** Representative images of CD68 labeling in the OSL (ROI indicated by white dashed line) of young, middle-aged, and aged mice. Note: occasionally the tectorial membrane was not completely removed adding noise in the signal. This noise was excluded from ROI analysis (see ROI in panel **C**). Scale bar = 200 μm. Arrows: indicate Iba1+ CD68^+^ macrophages. Triangles: indicate CD68 outside Iba1^+^ macrophages. **(D)** Quantification of CD68 shows significant increases across aging. *p < 0.05. **(E)** Co-localization of CD68 and Iba1 shows a trend toward increases in intracellular CD68 inside Iba1-labeled macrophages across aging. ^#^*p* < 0.1. **(F)** Quantification of CD68 outside Iba1-labeled macrophages shows an increase in Iba1^−^ CD68^+^ only in aged mice possibly indicating increased infiltration. *p < 0.05. ns: not significant.

Inflammaging describes the phenomenon of increased innate immune responses during normal aging and is associated with a decrease in the adaptive arm of the immune system (Finch, 2011; Chambers et al., 2021). This low-grade, chronic inflammation in various tissues throughout the body during aging contributes to the pathology of numerous age-related diseases (Finch, 2011). It is conceivable that the activation of cochlear macrophages in the OSL during normal aging may be associated with the progression of ARHL. Given the various kinds and degrees of macrophage activation (Mosser and Edwards, 2008), we chose to investigate a common marker of macrophage activation—CD68—which is a lysosomal marker that is elevated during increased phagocytic activity and corresponds with increased expression of canonical pro-inflammatory pathways of resident macrophages (Holness and Simmons, 1993; Raggi et al., 2017; Lin et al., 2019). We found that CD68 area increased significantly across the lifespan in OSL (Figures 2A–C; quantified in Figure 2D; CD68 mean area: Young = 0.43 ± 0.09, Middle-aged = 0.82 ± 0.11, Aged = 1.76 ± 0.39 μm^2^/100 μm^2^; *n* = 4 or 5; one-way ANOVA: *F*_(2,10)_ = 6.54, *p* < 0.05). Specifically, we found that CD68 was expressed both in Iba1-labeled macrophages (Figures 2A–C; arrows) and other cells not labeled by Iba1 (triangles). The average amount of CD68 within macrophages trended upward with aging in the OSL (Figure 2E; CD68^+^ Iba1^+^ area: Young = 1.50 ± 0.41, Middle-aged = 2.42 ± 0.72, Aged = 3.72 ± 0.68 μm^2^/100 μm^2^ Iba1 area; *n* = 4 or 5; one-way ANOVA: *F*_(2,10)_ = 3.19, *p* < 0.1). Similarly, CD68 expression in other cells was significantly increased during aging (Figure 2F; Iba1^−^ CD68^+^ area: Young = 56.31 ± 5.39, Middle-aged = 58.76 ± 3.22, Aged = 70.98 ± 2.60 μm^2^/100 μm^2^; *n* = 4 or 5; one-way ANOVA: *F*_(2,10)_ = 4.66, *p* < 0.05; Tukey’s multiple comparisons tests: *p* < 0.05). It suggests that during aging, resident macrophages in the OSL are activated with increased phagocytic activity. In addition, it is likely that the accumulation and recruitment of other immune cells may also play an important role in the progression of ARHL. The increased phagocytic activity at middle-age prior to significant hair cell loss indicates that inflammaging at the early stages of ARHL involves alternative stimuli, including possibly decreased activity or demyelination of nerve fibers, loss of synapses, and/or retraction of peripheral projections of SGNs.

### Microglia Become Activated in the CN During Aging

**Figure 3:**
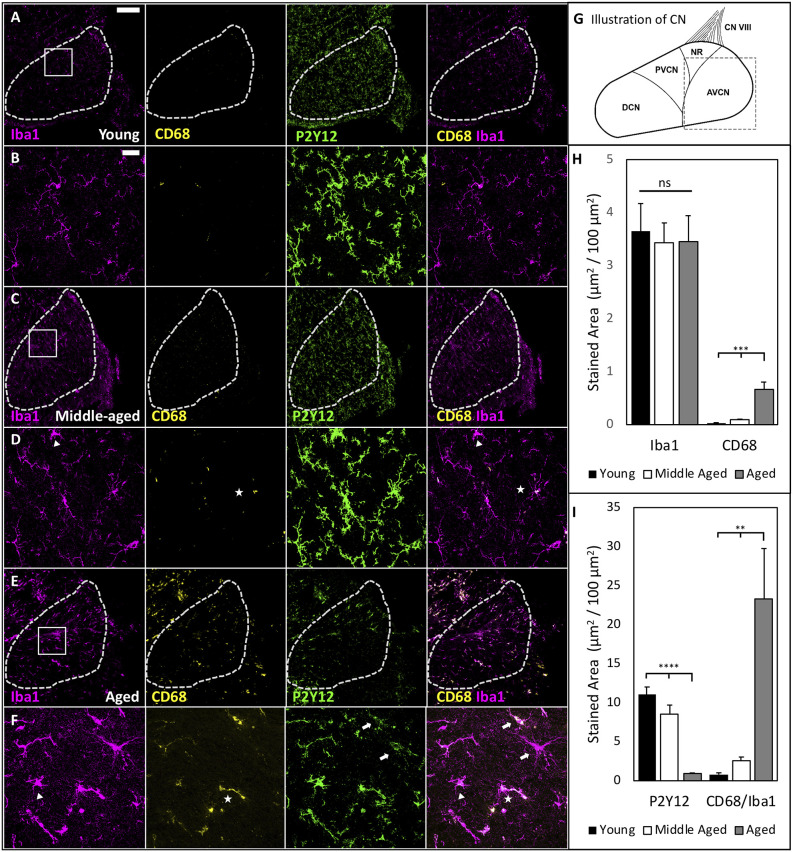
Age-associated microglia activation in the CN. **(A)** Representative images showing the staining of Iba1, CD68, P2Y12, and the overlapped image of Iba1 and CD68 in the AVCN of young mice. Dashed line marks the AVCN area for analysis; scale bar: 200 μm. **(B)** Magnified view of the ROI from **(A)**; scale bar: 25 μm. **(C–F)** Same images obtained from middle-aged mice **(C,D)** and aged mice **(E,F)**. Triangles mark: microglia with activation-associated morphology; arrows: microglia with major downregulation P2Y12; and stars: microglia with increased CD68 content. Importantly, these three approaches to identify activation capture various populations, suggesting heterogeneity of microglial activation in aged mice. Notice, for example, one of two microglia with downregulated P2Y12 (top arrow) also had increased CD68 staining, while the other does not. **(G)** Schematic of the CN. Dashed square mark AVCN as shown in **(A,C,E)**. **(H)** Summary changes of Iba1- and CD68-labeled area during aging. Iba1-labeled area did not significantly differ between groups, while CD68 increased with age. One-way ANOVA: ***p < 0.001. ns: not significant. **(I)** P2Y12 is downregulated during late-stage ARHL when the most dramatic increase in phagocytic activation occurs as quantified by colocalized area μm^2^/100 μm^2^ CD68/Iba1. One-way ANOVA: **p < 0.01; ****p < 0.0001.

Our previous studies in the CN, the first nucleus of the central auditory system, showed that auditory input to the brain is significantly reduced during ARHL (Xie, 2016; Xie and Manis, 2017). Given the ability of microglia to react to changes in sensory-driven neuronal activity (Tremblay et al., 2012), we further tested whether microglial activation occurs in the CN during ARHL. Parasagittal tissue sections containing the CN were fixed and stained by Iba1 and CD68 to investigate microglial activation during aging (Figure 3). Representative images from young, middle-aged, and aged CN (Figures 3A–F) show low and high magnification images of the anteroventral CN (AVCN; see schematic Figure 3G), where neurons receive AN input with ARHL-associated synaptopathy during normal aging (Wang et al., 2021). Compared to young mice, we found examples of microglia with ameboid morphology in middle-aged and aged mice (Figures 3D,F; triangle), suggesting microglia activation in the CN during the development of ARHL. However, no significant difference was found in the total Iba1-labeled area in the AVCN (Figure 3H), likely due to a balance between reduced numbers of microglia while each cell increased in area. CD68-labeling predominantly co-localized with Iba1-labeling across all age groups (Figures 3D,F; star), and was used to assess functional activation of microglia. The total CD68 area was very low in young mice, but significantly increased during aging (Figure 3H, CD68 area: Young = 0.029 ± 0.009, Middle-aged = 0.086 ± 0.017 and aged = 0.658 ± 0.145 μm^2^/100 μm^2^; *n* = 6 or 7; one-way ANOVA: *F*_(2,16)_ = 18.35, *p* < 0.0001; Tukey’s multiple comparisons tests: *p* < 0.001). Microglial activation, like macrophages, occurs on a spectrum and is best studied through multiple markers. P2Y12, a purinergic receptor uniquely expressed by microglia that is downregulated during activation (Zrzavy et al., 2017; Walker et al., 2020), can also provide insight into the state of microglia. We stained our samples with P2Y12 (Figure 3 green panels) and found that P2Y12 was significantly decreased in the AVCN of aged mice (Figure 3I, P2Y12 area: Young = 11.05 ± 0.96, Middle-aged = 8.59 ± 1.11 Aged = 0.60 ± 0.11 μm^2^/100 μm^2^; *n* = 6 or 7; one-way ANOVA: *F*_(2,16)_ = 34.13, *p* < 0.0001; Tukey’s multiple comparisons tests: *p* < 0.0001). High-resolution imaging revealed that the decrease in P2Y12 involves global downregulation in a heterogeneous manner, where the signal was significantly lost in some microglia (Figure 3F, arrows) but not others (Figure 3F, triangle, and star). Finally, we found that CD68 area, relative to Iba1 area, is mildly increased in middle-aged and drastically increased in aged mice (Figure 3I, CD68/IBA ratio: Young = 0.80 ± 0.22, Middle-aged = 2.58 ± 0.46, Aged = 23.26 ± 6.49 μm^2^/100 μm^2^; *n* = 6 or 7; one-way ANOVA: *F*_(2,16)_= 11.98, *p* < 0.001; Tukey’s multiple comparisons tests: *p* < 0.01). The results suggest that major microglial activation occurs following the substantial loss of input from the cochlea during late-stage ARHL.

### Complement Deposition in the Cochlear Nucleus Increases Dramatically During Aging

**Figure 4:**
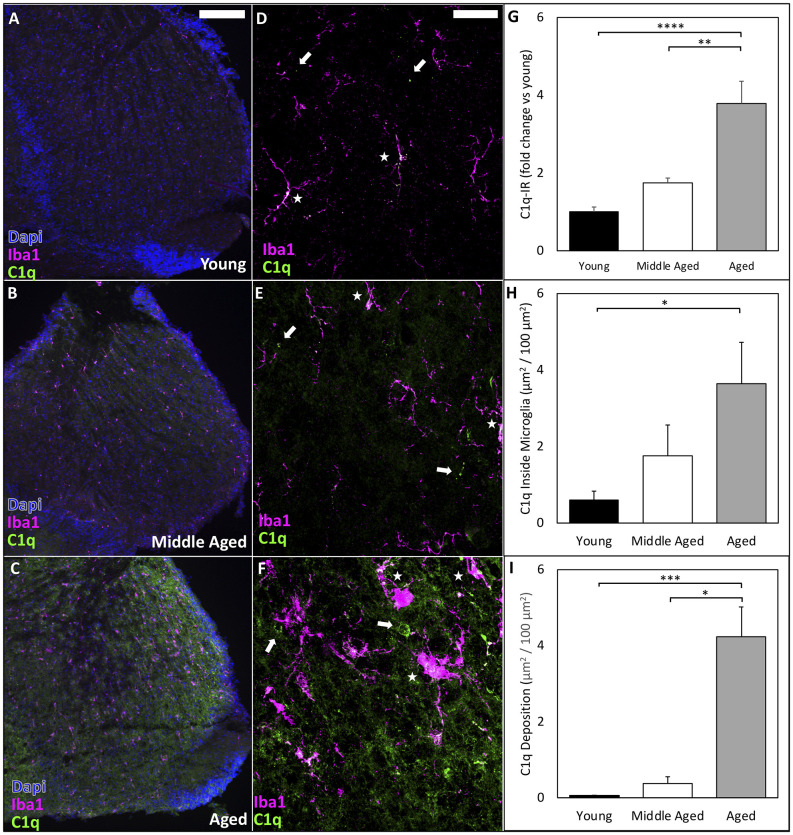
C1q deposition in the CN dramatically increases during ARHL. **(A–C)** Representative images to show that C1q-IR is progressively increased throughout the CN during aging. Scale bar = 200 μm. **(D–F)** High resolution confocal imaging reveals intracellular C1q inside microglia dramatically increases in aging. Arrows: C1q deposited on tissue outside of microglia; Stars: internal C1q inside microglia. Scale bar = 25 μm. **(G)** Quantification of C1q-IR by mean fluorescence intensity analyzed by ImageJ shows significantly increased C1q-IR across the lifespan. **p < 0.01; ****p < 0.0001. **(H)** C1q levels rise inside microglia across aging. *p < 0.05. **(I)** The total amount of C1q deposition increases with age. *p < 0.05; ***p < 0.001.

Complement deposition in neural tissues is associated with neuronal degeneration and increases with aging. C1q initiates the classical complement cascade and is typically used by the immune system for host defense and debris clearance following injury. However, C1q and the complement cascade also sculpt neural circuitry through synapse elimination during development and disease (Schafer et al., 2012; Hong et al., 2016a, b). We therefore labeled C1q in the AVCN and found significant increases in C1q immunoreactivity [C1q-IR (Stephan et al., 2013)] during aging (representative images shown in Figures 4A–F; quantified in Figure 4G; C1q-IR means: Young = 1.00 ± 0.13, Middle-aged = 1.75 ± 0.12, Aged = 3.79 ± 0.57 mean fluorescent intensity fold change vs. young; one-way ANOVA: *F*_(2,16)_ = 18.35, *p* < 0.0001; Tukey’s multiple comparisons tests: *p* < 0.01, *p* < 0.0001). We also observed intracellular C1q inside microglia dramatically increased across the lifespan (Figure 4H; C1q inside microglia: Young = 0.6 ± 0.23, Middle-aged = 1.75 ± 0.81, Aged = 3.64 ± 1.08 μm^2^/100 μm^2^; *n* = 6 or 7; Kruskal-Wallis: *H*_(3,19)_ = 7.122, *p* < 0.05; Dunn’s multiple comparisons tests: *p* < 0.05). Finally, we analyzed the content of C1q deposited onto the tissue in the AVCN (outside of microglia) and found significant increases across aging, with the most dramatic increase in aged mice (Figure 4I, C1q deposition: Young = 0.052 ± 0.014, Middle-aged = 0.375 ± 0.178, Aged = 4.24 ± 0.78 μm^2^/100 μm^2^; *n* = 6 or 7; Kruskal-Wallis: *H*_(3,19)_ = 13.62, *p* < 0.0001; Dunn’s multiple comparisons: *p* < 0.05, *p* < 0.001). These results demonstrate that the activation of microglia in the CN is accompanied by elevated production and tissue deposition of C1q. It suggests that neuroinflammation in the CN is significantly elevated during aging, in which microglial activation and complement deposition may contribute to the progression of ARHL.

## Discussion

### Innate Immune Activity Changes in the Auditory System Across the Lifespan

The cochlea, despite early identification as an immune privileged zone (Harris, 1983), can elicit immune functions both locally and in response to systemic immune challenges (Cai et al., 2014; Yang et al., 2016; Watson et al., 2017). Recent studies of the unique populations of immune cells in the cochlea have shown local immune cells and infiltrates (Wood and Zuo, 2017) from the blood do indeed participate in surveillance and host defense across the blood-labyrinth border (BLB). During normal aging, however, many changes occur including increased BLB permeability and baseline activation of innate immune function. These age-related changes can result in the accumulation of infiltrating immune cells and increased inflammation in tissues, including the cochlea and brain (Shi, 2016; Nyberg et al., 2019), leading to, or exacerbating, age-related pathology (Sun and Wang, 2015; Neng and Shi, 2020).

### Cochlea Inflammation Occur Prior to Severe ARHL

Inflammation was reported in the cochlea in humans with ARHL (Noble et al., 2019) and in animal models of early-onset ARHL (Su et al., 2020). In this study, we investigated the progression of chronic inflammation during normal aging in CBA/CaJ mice with human-like ARHL (Sergeyenko et al., 2013; Wang et al., 2021). We found that the accumulation of macrophages (Mosser and Edwards, 2008) correlates with the severity of hearing loss during normal aging, and that mild cochlear inflammation emerges at middle-age prior to the rise of severe ARHL with a profound elevation of hearing threshold (Figure 1F). Studies showed that at this early stage of ARHL (~18 months in CBA/CaJ mice), there is minimal loss of hair cells and SGNs (Sergeyenko et al., 2013), but significant loss of cochlear synapses and degeneration of AN central synapses (Wang et al., 2021). Therefore, it is likely that the accumulation and activation of cochlear macrophages in the OSL is not a result of cell death but rather changes in the activity of the sensory apparatus, including loss of synapses, retraction of peripheral processes of SGNs, and/or demyelination of AN fibers. Our findings suggest that there could be a window at the middle-age for therapeutic interventions in treating ARHL and that preventing the accumulation of macrophages early in disease progression is a promising target.

### Decreased Sensory Input May Drive Increased Complement Deposition in the AVCN

Our studies showed that AN synaptopathy in the CN contributes to functional deficits in ARHL (Xie, 2016; Xie and Manis, 2017; Wang et al., 2021). The mechanisms of such AN central synaptopathy remain unclear. It is known that neuroinflammation is involved in synaptic pruning during both development and disease, making it a tantalizing target for investigation. In addition to potential neuronal mechanisms for ARHL-associated synaptopathy, increased activation of brainstem microglia may contribute to the degradation of auditory synapses in the AVCN. Our findings that C1q significantly increases in the CN during aging suggest that the complement system is activated during the development of ARHL. Middle-aged mice showed smaller increases in microglial production and tissue deposited C1q suggesting this could be a window for therapeutic interventions. Further studies should examine the localization of C1q deposition and other components of the classical complement cascade on endbulb of Held synapses, and other central auditory synapses, during ARHL. Future interventions targeting innate immune pathways may provide exciting new opportunities for the development of novel therapeutics to prevent the progression of or restore lost function in ARHL.

## Data Availability Statement

The original contributions presented in the study are included in the article, further inquiries can be directed to the corresponding author.

## Ethics Statement

The animal study was reviewed and approved by Institutional Animal Care and Use Committee of The Ohio State University.

## Author Contributions

BS designed experiments, collected and analyzed data, and wrote the manuscript. SL and RX both assisted in data collection. RX provided oversight of experimental design. All authors contributed to the article and approved the submitted version.

## Conflict of Interest

The authors declare that the research was conducted in the absence of any commercial or financial relationships that could be construed as a potential conflict of interest.

## Publisher’s Note

All claims expressed in this article are solely those of the authors and do not necessarily represent those of their affiliated organizations, or those of the publisher, the editors and the reviewers. Any product that may be evaluated in this article, or claim that may be made by its manufacturer, is not guaranteed or endorsed by the publisher.
